# Correlation between *HER2* Expression and Clinicopathological Features of Breast Cancer: A Cross- Sectional Study in Vietnam

**DOI:** 10.31557/APJCP.2020.21.4.1135

**Published:** 2020-04

**Authors:** Thuan Dang Cong, Tung Nguyen Thanh, Quynh Anh Nguyen Phan, Ai Phuong Hoang Thi, Bao Song Nguyen Tran, Quoc Huy Nguyen Vu

**Affiliations:** 1 *Department of Histology, Embryology, Pathology and Forensic, *; 2 *Institute of Biomedical Research, *; 3 *Faculty of Basic Science, *; 4 *Department of Obstetrics and Gynaecology, Hue University of Medicine and Pharmacy, Hue University, Vietnam. *

**Keywords:** Breast cancer, HER2, immunohistochemistry, DISH, clinicopathological features

## Abstract

**Background:**

*HER2* is the target of the therapeutic agents which are used to treat HER2-positive breast cancer. Reports have shown that the *HER2* oncogene expression and its association with clinicopathological factors remain unclear in breast cancer (BC) patients. This study aimed to determine the correlation between *HER2 *expression and clinicalpathological characteristics of breast cancer in Vietnamese women.

**Methods:**

Between June 2016 and August 2018, paraffin-embedded specimens from 237 patients with primary invasive breast carcinoma in Hue University Hospital and Hue Center Hospital, Hue city, Vietnam were examined for pathological features. The gene expression of *HER2*, *ER*, *PR* and *Ki-67* were determined by immunohistochemistry (IHC). The gene amplification of *Her2* was assessed by using Dual color in situ hybridization (DISH).

**Results:**

The most frequent histological type was invasive carcinoma of no special type (NST) with 77.35%, the highest percentage of patients with Grade II was detected (59.36%), tumor size > 2 cm accounted for 71.31% of cases, Lymph node metastases were available in 57.86% cases. Most patients were diagnosed at stage II (59.18%). The majority of patients were classified as moderate Nottingham prognostic index (54.9%). Estrogen receptor and Progesterone receptor were positive in 53.16% and 50.63%, respectively. 76.37% of cases were in high expression group of Ki-67 (≥14%). HER2 IHC 2+, 3+ were accounted for 28.69% and *HER2* gene amplification was detected in 31% cases. *HER2* gene amplification and/or overexpression was significantly associated with cell proliferation index Ki67. Furthermore, *HER2* gene expression tended to be more frequently found in tumors with large tumor size, high grade, high stage and high Nottingham prognostic index and confirmed their prognostic independent role.

**Conclusions:**

Our data indicated that *HER2* gene expression was significantly correlated with cell proliferation index Ki67, but not significantly associated with another clinicopathological factors in breast cancer of Vietnamese women.

## Introduction

In women, breast cancer is considered to be the main cause of women’s deaths worldwide (Bray et al., 2018). There were more than two million newly diagnosed as breast cancer in women worldwide in 2018 (Bray et al., 2018; Jenkins et al. 2018).There will be an estimated 18.1 million new cancer cases and 9.6 million cancer deaths in 2018. In Vietnam, the incidence of breast cancer has been increasing steadily, from 13.8 per 100,000 women in 2000 to 29.9 per 100,000 women in 2010 and tends to increase year by year to 12,533 cases (Dieu et al., 2012; Jenkins Chris et al., 2019).

The proto-oncogene HER2/neu (C-erbB-2) has been localized to chromosome 17q21.1 and encodes a transmembrane tyrosine kinase growth factor receptor (Muleris et al., 1997). HER2 (human epidermal growth factor receptor 2) is a member of the epidermal growth factor receptor family, encodes a 185 kDa transmembrane glycoprotein with tyrosine kinase activity (Saini et al., 2011). Most studies on *HER2*-found this gene was involved in inducing mammary carcinogenesis. *HER2/neu* gene amplification has been associated with the development of breast cancer (Piechocki et al., 2007).

Several HER2-targeted therapeutic strategies have potential to adopt the *HER2* overexpression tumor cells: monoclonal antibodies downregulate* HER2 *expression by binding to the extracellular domain (such as trastuzumab and pertuzumab); antibody-drug conjugates (such as trastuzumab emtansine, called T-DM1); and tyrosine kinase inhibitors (such as lapatinib and neratinib), which compete for ATP-binding to block HER2 signaling (Di Modica et al., 2017). Metastatic HER2-positive breast cancer patients receiving HER2-targeted therapy typically experience better outcomes and improved prognosis than those with HER2-negative disease (Wilson et al., 2018). Accurate evaluation of HER2 status is essential for the management of patients eligible for HER2-targeting therapy. Therefore, overexpression of HER2 protein or gene amplification has been recognized as a prognostic factor of breast cancer.

Gene amplification and protein overexpression of *HER2* are found in approximately 15%- 20% of BC (Varga et al., 2013). IHC method of HER2 protein detection is advantageous, convenient, inexpensive, and only requires conventional microscopy. However, the results may be influenced by the time and the fixation protocol, the antibody clone, and most importantly it is difficult to apply the score sheet to have accurate conclusions. Therefore, for an ambiguous result obtained by immunohistochemical staining, the exact state of *HER2* gene amplification should be assessed by performing fluorescent in situ hybridization (FISH) or dual chromogenic in-situ hybridization (DISH) because of its high accuracy and reliability, although expensive (Ellis et al., 2005).

The correlation between *HER2* gene expression and breast cancer is not clear. According to most studies, it is thought that *HER2* overexpression is a poor prognostic factor (Allred et al., 1992). The breast cancer parameters such as lymph node metastases, tumor grade, cell proliferation were reported that associated with the gene expression of *HER2* (Aman N. A. et al., 2019a; Pengfei et al. 2019). In the initial reports, HER2/neu amplification was an important predictor of early relapse and death in breast cancer. *HER2* gene amplification has a significant predictor of both disease-free survival and time to relapse in breast cancer patients (Iqbal and Iqbal, 2014; Parkes et al., 2010a). However, several authors demonstrated that amplification of *HER2* gene relate to ER, PR status but not correlate with age, tumor size and lymph node (Shokouh et al., 2015). 

HER2 served as a poor prognostic indicatory but good predictor for response to anti HER2 therapy. Therefore, it is really meaningful to investigate correlation between HER2 expression and other clinicoopathologic parameters in Vietnamese women.

## Materials and Methods

*Patients and specimens*

The cross-sectional study was conducted on patients with primary invasive breast carcinomas in Hue University Hospital and Hue Center Hospital, Hue city, Vietnam from June 2016 to August 2018. The specimens were obtained from surgical resection of patients diagnosed with primary breast cancer, which have not been treated with pre-operative chemotherapy in the past yet. Patient’s information were extracted using a checklist including age, menopausal status, site, histological type, grade, stage, lymph node involvement and status of ER, PR, HER2, Ki-67. The status of ER, PR, HER2 and Ki-67 were determined by IHC upon the formalin fixed paraffin-embedded blocks of breast carcinoma patients.

All specimens were processed for histopathologic examination according to WHO Classification (Frank et al., 2013) by using hematoxylin and eosin staining and graded with the criteria of Elston and Ellis (2002).

*Immunohistochemistry staining*

The immunohistochemical staining was conducted on 3 μm thicknesses of breast tissue sections on the automated VENTANA BenchMark ULTRA platform (Ventana Medical Systems Inc., Tucson, AZ, USA) using antibodies: anti–Estrogen receptors (SP1), anti-Progesterone Receptor (1E2), anti-HER2/neu (4B5), anti-Ki-67 (30-9). IHC results were evaluated under light microscope. The Estrogen receptor (ER), Progesterone receptor (PR) staining results were scored as positive when greater than or equal to 1% of tumor cell nuclei were stained. Immunohistochemical analysis of the tumor proliferation index marker Ki-67 was performed using a cut-off point of 14%. HER2 protein expression were assessed based on intensity cell membrane immunostaining and the percentage of membrane positive cells. HER2 expression were classified from 0 to 3+; IHC staining 0, with absence or < 10% of invasive tumor cells with faint staining; IHC staining 1+, with > 10% of invasive tumor with faint incomplete membrane staining; IHC 2+, with > 10% of invasive tumor cells with incomplete or week membrane staining or ≤ 10% of invasive tumor cells with intense membrane staining; IHC 3+, with >10 % of invasive tumor cells with uniform intense membrane staining.

*Dual color in situ hybridization*

*HER2* gene amplification was defined by dual color in situ hybridization using the inform HER2 dual in situ hybridization DNA probed cocktail assay on the automated VENTANA BenchMark ULTRA platform (Ventana Medical Systems Inc., Tucson, AZ, USA). DNA Probe Cocktail assay is designed to determine *HER2 *gene status by detecting HER2 copies via silver in situ hybridization (SISH) (black) and chromosome 17 copies via chromogenic Red in situ hybridization (Red ISH) on a single slide. Pathologists scored the DISH studies, counting at least 20 tumor cells. The criteria consist of a combination of the HER2/CEP17 ratio and the average number of HER2 signals per cell. The *HER2* gene amplification was scored as “amplified” if the case had a HER2/CEP17 signal count ratio of 2.0 or if the HER2/CEP17 signal count ratio was <2.0 but the average number of HER2 signals per cell were 6.0. A score of “equivocal” was given if the case had a HER2/CEP17 signal count ratio of <2.0 and the average number of HER2 signals per cell were ≥ 4.0 and <6.0. A score of “not amplified” was given if the case had a HER2/CEP17 signal count ratio of <2.0 and the average number of HER2 signals were <4.0 (Nishimura et al., 2016a; Nishimura et al., 2016b).

*Statistical analysis*

The data was analyzed using R and Statistical Package for Social Sciences (SPSS, Ver 17). Chi square tested was used to test the relationships between the clinicopathological factors and *HER2 *expression. A p-value of less than 0.05 was considered statistically significant.

## Results

*Patient and tumour characteristics of breast cancer patients*

The clinicopathologic characteristics of breast cancer patients are presented in Table 1 and Supplementary Figure 1. The majority of patients were older than 40 years of age (54.70 ± 12.66) and location of breast tumor was different, in which the upper outer quadrant was the most frequent site (54.69%). The predominant histological type was invasive carcinoma of no special type (NST) with 181 cases (77.35%). The significant difference was found when subtypes were compared to histological grade. The highest percentage of patients with Grade II was detected (59.36%). Median tumor diameter was 3.37 ± 1.83 cm (27.16% cases ≤ 2cm and 72.84% case > 2cm). Lymph node metastases were available in 57.86% cases (68 cases with 1-3 lymph nodes metastases and 46 cases have over 4 lymph nodes metastases) and 42.13% of cases with no lymph nodes available (83 cases). There were significant differences in the distribution of stage of the breast tumors. In Supplementary Figure 1, the breast cancer staging was 11.22% for stage I, 59.18% for stage II, 29.60% for stage III. The Nottingham prognostic index (NPI) is an established prognostication tool for the management of breast cancers (Fong et al., 2015). The majority of patients were classified as moderate Nottingham prognostic index (107 patients, 54.9%).

*Molecular characteristics of patients with breast cancer*

**Figure 1:**
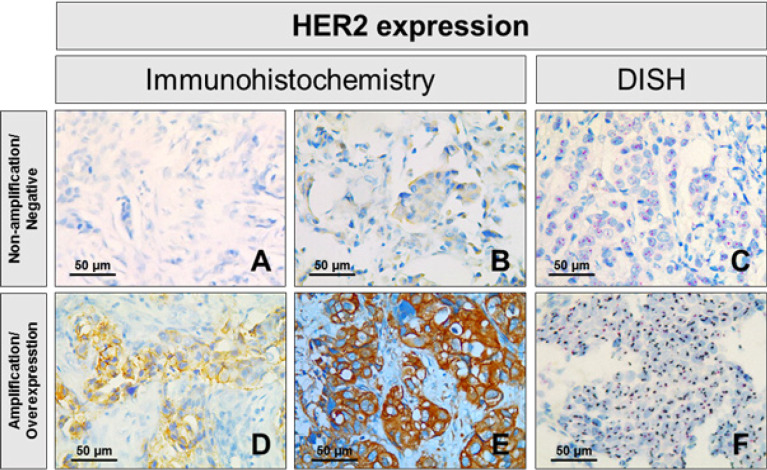
Immunohistochemical (IHC) and Dual Color in Situ Hybridization (DISH) Analysis of Human Epidermal Growth Factor Receptor 2 (HER2) in Breast Cancer. A. Immunohistochemical score 0: No staining on tumor cell membrane; B. Immunohistochemical score 1: Faintly perceptible staining on > 10% tumor cell membrane; C. Case without HER2 amplification using DISH analysis; D. Immunohistochemical score 2: Moderate staining on > 10% tumor cell membrane; E. Immunohistochemical score 3: Strong staining on > 10% tumor cell membrane; F. Case with HER2 amplification using DISH analysis. Pink color refer to the reference probe of Chr. 17 centromere, while brown color are the target probe for HER2

**Figure 2:**
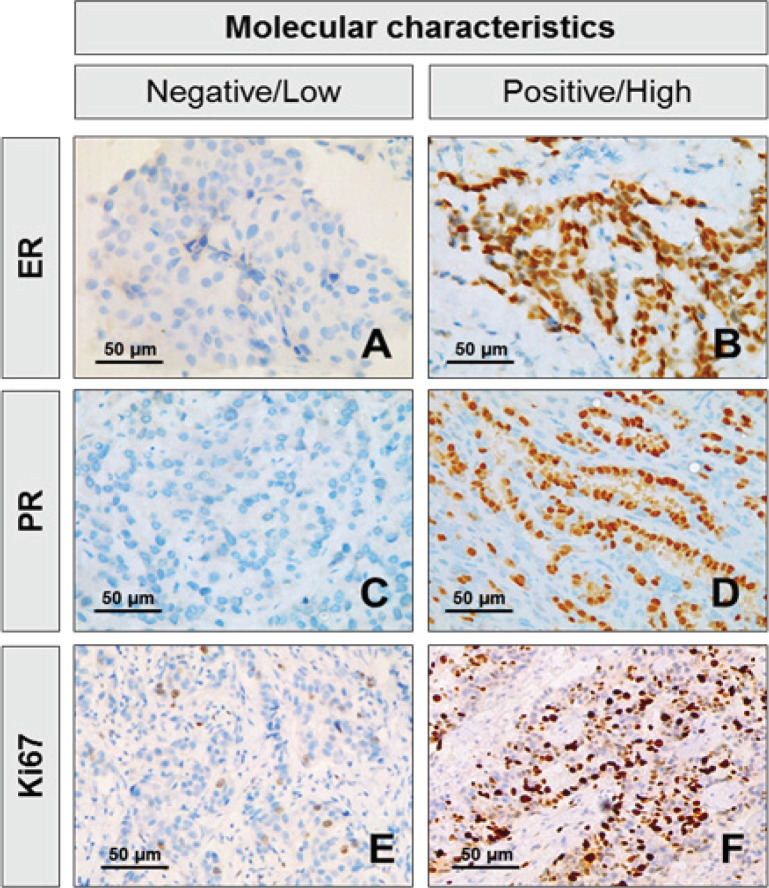
Immunohistochemical Characteristics of ER, PR, HER2 and Ki-67 Markers in the Tumor Sample from Breast Cancer. A. Estrogen receptor negative; B. Estrogen receptor positive; C. Progesterone receptor negative; D. Progesterone receptor positive; E. Low expression of Ki-67 (≥14%) ; F. High expression of Ki-67 (≥14%)

The molecular characteristics of breast cancer patients are summarized in Table 2 and Supplementary Figure 2. *HER2* gene amplification status was estimated by DISH (Figure 1). In 100 cases DISH analyzed, 31 cases showed the result of the amplification of *HER2* gene in breast cancer. Overexpression of HER2 protein of breast cancer tissues was accessed by IHC (Figure 1). In 237 cases, 68 cases (28.69%) were scored as HER2 IHC positive (2+, 3+), the remaining 169 cases were HER2 IHC negative (71.31%). Estrogen receptor status was positive about 126 cases (53.16%) while Progesterone receptor was positive in 120 cases (50.63%). The expression of proliferation marker Ki-67 differed significantly from the subtypes (p<0.001). There were 181 cases (76.37%) with high expression of Ki-67 (≥14%), while its low expression (<14%) was detected in 56 cases (23.63%) (Figure 2). The molecular classification of breast cancer cases were statistically significant difference. Statistically significant difference was observed in the molecular classification of breast cancer cases. The majority of patients were classified as Luminal B HER2-negative (32.07%). Compared with luminal A cases, triple negative and *HER2*-overexpression were more likely to be poorly differentiated at diagnosis. On the basis of histological examination, Luminal A showed the percentage of cases with low expression of Ki-67 was 18.57% while *HER2*-overexpression and triple negative were 16.88% and 21.52%, respectively.

*Correlation of HER2 protein overexpression with clinicopathological features*

Correlation of HER2 protein overexpression with clinicopathological features was showed in Table 3 and Supplementary Figure 3.* HER2* overexpression was significantly associated with cell proliferation index. There were no statistically significant associations between *HER2* overexpression and tumor size, histological grade, stage, lymph node metastases, Nottingham prognostic index. However, there was a trend toward increasing *HER2* overexpression in tumor having larger size, higher grade, higher stage and high Nottingham prognostic index (Supplementary Figure 3).

*Correlation of HER2 gene amplification with clinicopathological features*

Correlation of *HER2* gene amplification detected by DISH with clinicopathological features are presented in Table 4 and Supplementary Figure 4. In our study, statistical analysis showed a significant correlation between *HER2* gene amplification and proliferation index Ki-67 (p = 0.024). However, the *HER2* gene amplification was not significantly related to other clinicopathological features including tumor size, histological grade, lymph node metastases, breast cancer staging and Nottingham prognostic index.

**Table 1:** Patient and Tumour Characteristics of Patients with Breast Cancer

Characteristics	n (%)
Age (54.70 ± 12.66)	
<30	2 (0.8)
30-39	21 (8.9)
40-49	64 (27)
50-59	77 (32.5)
>60	73 (30.8)
Periods	
Having periods	103 (43.5)
Menopause	134 (56.5)
Location of breast tumours	
Upper outer quadrant	70 (54.69)
Upper inner quadrant	30 (23.44)
Lower outer quadrant	13 (10.16)
Lower inner quadrant	5 (3.91)
Central	10 (7.81)
Histological classification	
Invasive carcinoma of no special type (NST)	181 (77.35)
Invasive lobular carcinoma	24 (10.26)
Mixed lobular carcinoma	5 (2.14)
Metaplastic carcinoma of no special type	9 (3.84)
Mucinous carcinoma	12 (5.13)
Carcinoma with apocrine differentiation	2 (0.85)
Carcinoma with medullary features	1 (0.43)
Tumor size (3.37 ± 1.83 cm)	
≤ 2 cm	63 (27.16)
> 2 cm	169 (72.84)
Histological grade	
Grade I	22 (11.76)
Grade II	111 (59.36)
Grade III	54 (28.88)
Breast cancer staging	
Stage I	22 (11.22)
Stage IIA	51 (26.02)
Stage IIB	65 (33.16)
Stage IIIA	41 (20.92)
Stage IIIB	4 (2.04)
Stage IIIC	13 (6.63)
Lymph node metastases (2.54 ± 3.85)	
0	83 (42.13)
1-3	68 (34.51)
≥ 4	46 (23.35)
The Nottingham prognostic index (NPI)	
NPI I (Excellent) Score ≤2.4	21 (10.80)
NPI II (Good) Score >2.4 but ≤3.4	10 (5.10)
NPI III (Moderate) Score >3.4 but ≤5.4	107 (54.90)
NPI IV (Poor) Score >5.4	57 (29.20)

**Table 2:** Molecular Characteristics of Patients with Breast Cancer

Characteristics	n (%)	95% CI	*P*
HER2 dual in situ hybridization (DISH)			
Non-amplified	69 (69.00)	(25.17 - 37.31)	< 0.001
Amplified	31 (31.00)	(22.13 - 41.03)	
HER2 immunohistochemistry			
Negative (0, 1+)	169 (71.31)	(23.02 - 34.90)	< 0.001
Positive (2+, 3+)	68 (28.69)	(65.10 - 76.98)	
ER immunohistochemistry			
Negative	111 (46.84)	(40.35 - 53.41)	0.051
Positive	126 (53.16)	(46.59 - 59.65)	
PR immunohistochemistry			
Negative	117 (49.37)	(42.84 - 55.92)	0.698
Positive	120 (50.63)	(44.08 - 57.16)	
Cell proliferation index			
Low (Ki67<14%)	56 (23.63)	(18.37 - 29.56)	< 0.001
High (Ki67≥14%)	181 (76.37)	(70.44 - 81.63)	
Molecular classification			
Luminal A (ER+ or PR+; HER2-; Ki67 low)	44 (18.57)	(13.83 - 24.12)	< 0.001
Luminal B HER2-negative (ER+ or PR+; HER2-; Ki67 high)	76 (32.07)	(26.17 - 38.42)	
Luminal B HER2-positive (ER+ or PR+; HER2+; Ki67 any)	26 (10.97)	(7.29 - 15.66)	
HER2-overexpression (ER-; PR-; HER2+; Ki67 any)	40 (16.88)	(12.34 - 22.27)	
Triple negative (ER-; PR-; HER2-; Ki67 any)	51 (21.52)	(16.46 - 27.30)	

**Table 3:** Correlation between HER2 Protein Overexpression and Clinicopathological Characteristics in Breast Cancer Patients

Characteristics	HER2 IHC		*p*
	Negative n (%)	Positive n (%)	
Tumor size			
≤ 2 cm	47 (20.3)	16 (6.9)	0.587
> 2 cm	120 (51.7)	49 (21.1)	
Histological grade			
Grade I	16 (8.6)	6 (3.2)	0.282
Grade II	81 (43.3)	30 (16)	
Grade III	33 (17.6)	21 (11.2)	
Breast cancer staging			
Stage I	17 (8.7)	5 (2.6)	0.272
Stage II	84 (42.9)	32 (16.3)	
Stage III	36 (18.4)	22 (11.2)	
Lymph node metastases			
0	61 (31.0)	22 (11.2)	
1-3	48 (24.4)	20 (10.2)	0.459
≥ 4	29 (14.7)	17 (8.6)	
Cell proliferation index			
Low	49 (20.7)	7 (3.0)	0.002
High	120 (50.6)	61 (25.7)	
Nottingham prognostic index			
NPI I	16 (8.2)	5 (2.6)	0.77
NPI II	7 (3.6)	3 (1.5)	
NPI III	76 (39.0)	31 (15.9)	
NPI IV	37 (19.0)	20 (10.3)	

**Table 4:** Correlation between HER2 Gene Amplification and Clinicopathological Characteristics in Breast Cancer Patients

Characteristics	HER2 DISH		*P*
	Non-amplified n (%)	Amplified n (%)	
Tumor size			
≤ 2 cm	13 (13.0)	11 (11.0)	0.071
> 2 cm	56 (56.0)	20 (20.0)	
Histological grade			
Grade I	7 (8.6)	2 (2.5)	0.248
Grade II	38 (46.9)	15 (18.5)	
Grade III	10 (12.3)	9 (11.1)	
Breast cancer staging			
Stage I	7 (7.1)	4 (4.0)	0.929
Stage II	43 (43.4)	19 (19.2)	
Stage III	18 (18.2)	8 (8.1)	
Lymph node metastases			
0	29 (29.0)	11 (11.0)	0.766
1-3	26 (26.0)	12 (12.0)	
≥ 4	14 (14.0)	8 (8.0)	
Cell proliferation index			
Low	32 (32.0)	7 (7.0)	0.024
Hight	37 (37.0)	24 (24.0)	
Nottingham prognostic index			
NPI I	7 (7.1)	4 (4.0)	0.917
NPI II	1 (1.0)	1 (1.0)	
NPI III	42 (42.4)	18 (18.2)	
NPI IV	18 (18.2)	8 (8.1)	

## Discussion

In the case of breast cancer, HER2 is an important prognostic factor for both clinicians and patients, and it is confirmed to be a factor related to poor prognosis (Kaptain et al., 2001). With the present study we want to determine the first correlation between *HER2* expression and clinical characteristics of breast cancer in Central Vietnam patients.

HER2 amplified or overexpressed in approximate 15 - 20% of breast cancer and associated with aggressive clinical feature if absence therapy (Figueroa-Magalhaes et al., 2014; Krishnamurti and Silverman, 2014). *HER2 *gene amplification and protein over-expression are important not only as a prognostic factor but also as a predictive clinical response to anti-HER2 therapeutic (Parkes et al., 2010; Schneeweiss et al. 2014). Moreover, overall survival in patients treating with HER2-targeted therapy was higher than patients without treatment. (Mendes et al., 2015).

Determination of* HER2* gene status in breast cancer specimens has been performed at the *HER2* gene copies level using Polymerase chain reaction, Southern blotting, Fluorescence in situ hybridization (FISH), Chromogenic in situ hybridization (CISH) or Dual-colour chromogenic in situ hybridization (Dual-ISH or DISH) (Furrer et al., 2015). The DISH technique is an innovation of the FISH technique that allows observing and evaluating under bright-field microscope with the additional benefit that morphological features can be observed, and that possible storage slides at room temperature (Furrer et al., 2015). Therefore, DISH has indicated several advantages compared with the use of FISH. At the protein level, HER2 status was analyzed by using Western blotting, enzyme immunoassays (ELISA) or IHC technique (Furrer et al., 2015, Zaha 2014). Similar to DISH technique, IHC also had several advantages including the ability of assessment using a conventional bright-field microscope, tissue blocks and slides can be conveniently stored. However, IHC technique was affected by numerous factors such as the temperature, time of fixation, commercially available antibodies display different specificity and sensitivity (Furrer et al., 2015). Therefore, to exactly determine HER2 status in breast cancer, in situ hybridization should be performed that combined with IHC technique. Accurate assessment of HER2 status is essential for identification of breast cancer patients that may benefit from treatment with targeted HER2 therapy (Grant et al., 2015).

In recent studies, the authors have found correlation between the overexpression of* HER2* with the Nottingham grade, lymph node metastases (Aman et al., 2019; Bartlett et al., 2007). In addition, another authors indicated that gene amplification of the *HER2* oncogene was linked to the increase of tumor size, differentiation grade and TNM stage (Aman et al., 2019; Shi et al., 2019). Different data confirm previous reports that *HER2* overexpression in breast cancer is associated with ER-negative and PR-negative disease (Bartlett et al., 2007). This study revealed that the *HER2* overexpression was closely associated with cell proliferation index. The other clinicopathological features including tumor size, histological grade, stage, lymph node metastases, Nottingham prognostic index were not significant associated with amplification of* HER2* gene and *HER2* overexpression. However, the cases with* HER2* gene amplification and/or overexpression tended to be more frequently found in tumor with large tumour size, high grade, high stage and high Nottingham prognostic index.

HER2 is one of biomarkers that play an important role in breast cancer classification. Based on ER, PR, HER2, Ki-67 marker, breast cancer can be divided into 5 groups: Luminal A (ER+ or PR+; HER2-; Ki67 low), Luminal B HER2-negative (ER+ or PR+; HER2-; Ki67 high), Luminal B HER2-positive (ER+ or PR+; HER2+; Ki67 any), HER2-overexpression (ER-; PR-; HER2+; Ki67 any), Triple negative (ER-; PR-; HER2-; Ki67 any) (Onitilo et al., 2009). In addition, classification based on *HER2* expression provides enhanced and important therapeutic guidance (Iqbal and Iqbal, 2014). Patient with subtype absence *HER2* expression will have poor prognosis and not receive most benefit from chemotherapy (Knutson et al., 2016).

Previous several studies considered NPI as a clinically useful tool for stratification of patients by incorporating traditional prognostic factors tumor size, lymph node status and histological grade (Rakha et al., 2014). Combination HER2 status to NPI can be improved its predictive ability accuracy, especially in the intermediate prognostic category of NPI (Van Belle et al., 2010). In our study, *HER2* overexpression and amplification were not significant associated with Nottingham prognostic index. Nevertheless, *HER2* overexpression tended to be observed in high Nottingham prognostic index tumours.

Cell proliferation index (Ki67) is also a prognostic and predictive factor of response to chemotherapy in breast cancer (Penault-Llorca and Radosevic-Robin, 2017). In previous researches, the cases had tumors with a high proliferative activity added to* HER-2* overexpression indicated a risk of relapse approximately higher than patients with slowly proliferation (Cheang et al., 2009; Ribelles et al., 2013). In the present study, we revealed that the extent of *HER2* status expression was significant associated with cell proliferation index Ki67.

In conclusion, this study revealed that Her2 status (gene amplification and protein overexpression) in breast cancer of Vietnamese women was significantly associated with cell proliferation index Ki67 but not with other clinicopathological features including tumor size, histological grade, stage, lymph node metastases, Nottingham prognostic index. However, the cases with *HER2* gene amplification and/or overexpression tended to be more frequently found in tumor with large tumor size, high grade, high stage and high Nottingham prognostic index.

## References

[B1] A Frank G, Danilova N, Andreeva I, Oleynikova N (2013). WHO Classification of tumors of the breast, 2012.

[B2] Allred DC, Clark GM, Molina R (1992). Overexpression of HER-2/neu and its relationship with other prognostic factors change during the progression of in situ to invasive breast cancer. Hum Pathol.

[B3] Aman NA, Doukoure B, Koffi KD (2019a). HER2 overexpression and correlation with other significant clinicopathologic parameters in Ivorian breast cancer women. BMC Clin Cathol.

[B4] Aman NA, Doukoure B, Koffi KD (2019b). HER2 overexpression and correlation with other significant clinicopathologic parameters in Ivorian breast cancer women. BMC Clin Pathol.

[B5] Bartlett JM, Ellis IO, Dowsett M (2007). Human epidermal growth factor receptor 2 status correlates with lymph node involvement in patients with estrogen receptor (ER)–negative, but with grade in those with ER-positive early-stage breast cancer suitable for cytotoxic chemotherapy. J Clin Oncol.

[B6] Bray F, Ferlay J, Soerjomataram I (2018). Global cancer statistics (2018): GLOBOCAN estimates of incidence and mortality worldwide for 36 cancers in 185 countries. CA Cancer J Clin.

[B7] Cheang MC, Chia SK, Voduc D (2009). Ki67 index, HER2 status, and prognosis of patients with luminal B breast cancer. J Nat Cancer Instit.

[B8] Di Modica M, Tagliabue E, Triulzi T (2017). Predicting the efficacy of HER2-targeted therapies: A look at the host. Dis Markers.

[B9] Dieu B, Duc N, Thuan T (2012). Cancer challenges and national cancer control programs to 2020. Viet Nam J Oncol.

[B10] Ellis C, Dyson M, Stephenson T, Maltby E (2005). HER2 amplification status in breast cancer: a comparison between immunohistochemical staining and fluorescence in situ hybridisation using manual and automated quantitative image analysis scoring techniques. J Clin Pathol.

[B11] Elston CW, Ellis IO (2002). Pathological prognostic factors in breast cancer The value of histological grade in breast cancer: experience from a large study with long-term follow-up. Histopathology.

[B12] Figueroa-Magalhaes MC, Jelovac D, Connolly R, Wolff AC (2014). Treatment of HER2-positive breast cancer. Breast J.

[B13] Fong Y, Evans J, Brook D (2015). The Nottingham prognostic index: five- and ten-year data for all-cause survival within a screened population. Ann R Coll Surg Engl.

[B14] Furrer D, Sanschagrin F, Jacob S, Diorio C (2015). Advantages and disadvantages of technologies for HER2 testing in breast cancer specimens. Am J Clin Pathol.

[B15] Grant KA, Pienaar FM, Brundyn K (2015). Incorporating microarray assessment of HER2 status in clinical practice supports individualised therapy in early-stage breast cancer. Breast J.

[B16] Iqbal N, Iqbal N (2014). Human epidermal growth factor receptor 2 (HER2) in cancers: Overexpression and therapeutic implications. Mol Biol Int.

[B17] Jenkins C, Ngan TT, Ngoc NB (2019). Strengthening breast cancer services in Vietnam: a mixed-methods study. Global Health Res Plicy.

[B18] Jenkins C, Minh LN, Anh TT (2018). Breast cancer services in Vietnam: a scoping review. Glob Health Action.

[B19] Kaptain S, Tan LK, Chen B (2001). Her-2/neu and breast cancer. Diagn Mol Pathol.

[B20] Knutson KL, Clynes R, Shreeder B (2016). Improved survival of HER2+ breast cancer patients treated with trastuzumab and chemotherapy is associated with host antibody immunity against the HER2 intracellular domain. Cancer Res.

[B21] Krishnamurti U, Silverman JF (2014). HER2 in breast cancer: a review and update. Adv Anat Pathol.

[B22] Mendes D, Alves C, Afonso N (2015). The benefit of HER2-targeted therapies on overall survival of patients with metastatic HER2-positive breast cancer–a systematic review. Breast Cancer Res.

[B23] Muleris M, Almeida A, Malfoy B, Dutrillaux B (1997). Assignment of v-erb-b2 avian erythroblastic leukemia viral oncogene homolog 2 (ERBB2) to human chromosome band 17q21 1 by in situ hybridization. Cytogenetic Genome Res.

[B24] Nishimura R, Okamoto N, Satou M, Kojima K, Tanaka S (2016a). HER 2 immunohistochemistry for breast cancer cell blocks can be used in the same way as that used for histological specimens. Diagn Cytopathol.

[B25] Nishimura R, Okamoto N, Satou M (2016b). Bright-field HER2 dual in situ hybridization (DISH) assay on breast cancer cell blocks: a comparative study with histological sections. Breast Cancer.

[B26] Onitilo AA, Engel JM, Greenlee RT, Mukesh BN (2009). Breast cancer subtypes based on ER/PR and Her2 expression: comparison of clinicopathologic features and survival. Clin Med Res.

[B27] Parkes E, McKenna S, McAleer J (2010a). HER2 as a prognostic factor in metastatic breast cancer treated with taxanes. J Clin Oncol.

[B28] Penault-Llorca F, Radosevic-Robin N. (2017). Ki67 assessment in breast cancer: an update. Pathology.

[B29] Pengfei S, Cheng C, Yufeng Y (2019). Correlation between HER-2 gene amplification or protein expression and clinical pathological features of breast cancer. Cancer Biotherapy Radiopharmaceuticals.

[B30] Piechocki MP, Yoo GH, Dibbley SK, Lonardo F (2007). Breast cancer expressing the activated HER2/neu is sensitive to gefitinib in vitro and in vivo and acquires resistance through a novel point mutation in the HER2/neu. Cancer Res.

[B31] Rakha E, Soria D, Green AR (2014). Nottingham Prognostic Index Plus (NPI+): a modern clinical decision making tool in breast cancer. Br J Cancer.

[B32] Ribelles N, Perez-Villa L, Jerez JM (2013). Pattern of recurrence of early breast cancer is different according to intrinsic subtype and proliferation index. Breast Cancer Res.

[B33] Saini KS, Azim Jr HA, Metzger-Filho O (2011). Beyond trastuzumab: new treatment options for HER2-positive breast cancer. Breast J.

[B34] Schneeweiss A, Chia S, Hegg R (2014). Evaluating the predictive value of biomarkers for efficacy outcomes in response to pertuzumab-and trastuzumab-based therapy: an exploratory analysis of the TRYPHAENA study. Breast Cancer Res.

[B35] Shi P, Chen C, Yao Y (2019). Correlation between HER-2 gene amplification or protein expression and clinical pathological features of breast cancer. Cancer Biotherapy Radiopharmaceuticals.

[B36] Shokouh TZ, Ezatollah A, Barand P (2015). Interrelationships between Ki67, HER2/neu, p53, ER, and PR status and their associations with tumor grade and lymph node involvement in breast carcinoma subtypes: Retrospective-observational analytical study. Medicine.

[B37] Van Belle V, Van Calster B, Brouckaert O (2010). Qualitative assessment of the progesterone receptor and HER2 improves the Nottingham Prognostic Index up to 5 years after breast cancer diagnosis. J Clin Oncol.

[B38] Varga Z, Noske A, Ramach C, Padberg B, Moch H (2013). Assessment of HER2 status in breast cancer: overall positivity rate and accuracy by fluorescence in situ hybridization and immunohistochemistry in a single institution over 12 years: a quality control study. BMC Cancer.

[B39] Wilson FR, Coombes ME, Brezden-Masley C (2018). Herceptin(R) (trastuzumab) in HER2-positive early breast cancer: a systematic review and cumulative network meta-analysis. Syst Rev.

[B40] Zaha DC (2014). Significance of immunohistochemistry in breast cancer. World J Clin Oncol.

